# The voltage-gated proton channel Hv1 promotes microglia-astrocyte communication and neuropathic pain after peripheral nerve injury

**DOI:** 10.1186/s13041-021-00812-8

**Published:** 2021-06-28

**Authors:** Jiyun Peng, Min-Hee Yi, Heejin Jeong, Przemyslaw Peter McEwan, Jiaying Zheng, Gongxiong Wu, Shashank Ganatra, Yi Ren, Jason R. Richardson, Seog Bae Oh, Long-Jun Wu

**Affiliations:** 1 Department of Neurology, Mayo Clinic, Rochester, MN 55905 USA; 2 Department of Cell Biology and Neuroscience, Rutgers University, Piscataway, NJ 08854 USA; 3 Institute of Life Science, Nanchang University, Nanchang, 330031 China; 4 Dental Research Institute and Department of Neurobiology and Physiology, School of Dentistry, Seoul National University, Seoul, 03080 Republic of Korea; 5 One Harvard Street Institute of Health, Brookline, MA 02446 USA; 6 Department of Biomedical Sciences, Florida State University, Tallahassee, FL 32306 USA; 7 Departments of Environmental Health Sciences, Florida International University, Miami, FL 33199 USA; 8 Department of Neuroscience, Mayo Clinic, Jacksonville, FL 32224 USA; 9 Department of Immunology, Mayo Clinic, Rochester, MN 55905 USA

**Keywords:** Microglia, Hv1 proton channel, Hvcn1, Reactive oxygen species, IFN-γ, Microglia-astrocyte interaction, Peripheral nerve injury, Neuropathic pain

## Abstract

Activation of spinal cord microglia contributes to the development of peripheral nerve injury-induced neuropathic pain. However, the molecular mechanisms underlying microglial function in neuropathic pain are not fully understood. We identified that the voltage-gated proton channel Hv1, which is functionally expressed in spinal microglia, was significantly increased after spinal nerve transection (SNT). Hv1 mediated voltage-gated proton currents in spinal microglia and mice lacking Hv1 (Hv1 KO) display attenuated pain hypersensitivities after SNT compared with wildtype (WT) mice. In addition, microglial production of reactive oxygen species (ROS) and subsequent astrocyte activation in the spinal cord was reduced in Hv1 KO mice after SNT. Cytokine screening and immunostaining further revealed that IFN-γ expression was compromised in spinal astrocytes in Hv1 KO mice. These results demonstrate that Hv1 proton channel contributes to microglial ROS production, astrocyte activation, IFN-γ upregulation, and subsequent pain hypersensitivities after SNT. This study suggests Hv1-dependent microglia-astrocyte communication in pain hypersensitivities and identifies Hv1 as a novel therapeutic target for alleviating neuropathic pain.

## Introduction

Peripheral nerve injury often results in neuropathic pain, which persists even after the initial injury heals. Such hypersensitivities are typically manifest as increased sensitivity to normally painful (hyperalgesia) and normally painless (allodynia) stimuli. It is well accepted that neuropathic pain is due to pathological alterations of both peripheral and central nociceptive neural networks [1]. However, recent studies have shown that glial-neuronal interactions play a key role in central sensitization and neuropathic pain outcomes [2, 3]. In particular, there is sequential activation of neurons, microglia, and astrocytes in the spinal cord after peripheral nerve injury [4, 5].

Microglia, the resident immune cells in the central nervous system (CNS), are extremely sensitive to the brain microenvironment [6–9]. Peripheral nerve injuries reliably induce microglial activation, which is indicated by hypertrophic morphologies, proliferation, gene expression alterations, and release of cytokines/chemokines [3, 10]. Using microglia ablation and chemogenetic approaches, studies have shown that microglial proliferation and neuroinflammation in response to nerve injury are key players in the induction of pain hypersensitivities [11–15]. Interestingly, optogenetic activation of spinal microglia is able to directly trigger “microgliogenic pain” [16]. The most well-known molecular mechanism involves microglial P2X4 receptor (P2X4R), which induces BNDF release and dis-inhibition in spinal lamina I neurons leading to pain hyperalgesia [17–20]. Many microglial receptors and ion channels, including P2Y12R, P2X7R, CCR2, CX_3_CR1, and NADPH oxidase 2 (Nox2) are also reported to promote neuropathic pain [5, 21–23]. In addition to microglia, peripheral nerve injuries also induce spinal astrocyte activation with increased glial fibrillary acidic protein (GFAP) expression. The release of pro-inflammatory cytokines/chemokines from activated spinal astrocytes, such as IFN-γ, MCP-1, and CXCL1, is able to prolong hypersensitive pain state after peripheral nerve injuries [24].

Among inflammatory mediators released by microglia and astrocytes, reactive oxygen species (ROS) play critical roles in both the initiation and maintenance of chronic pain. ROS production is increased in the spinal cord after neuropathic pain and ROS scavengers or antioxidants attenuated neuropathic pain [22, 25–27]. Recent studies have revealed that ROS generated by NADPH oxidases (NOX), including Nox2 and Nox4, contribute to neuropathic pain [22, 25, 26]. Nox2-deficient mice show attenuated mechanical allodynia and thermal hyperalgesia as well as reduced ROS generation, microglial activation, and pro-inflammatory cytokine expression after peripheral nerve injury [22].

Hv1 is a voltage-gated proton channel that was cloned in 2006 [28, 29] and later found to be specifically expressed in microglia, but not astrocytes or neurons [30–32]. Evidence indicates that Hv1 is coupled to NOX-dependent acidosis, membrane depolarization, or ROS production in a variety of cells, including neutrophils [33], B cells [34], and eosinophils [35]. Previous studies demonstrated that microglial Hv1 proton channels contribute to neuronal cell death through NOX-dependent ROS production and brain damage after ischemic stroke, brain injury and demyelination [32, 36–40]. More recently, the Hv1 channel in spinal microglia was shown to contribute to spinal cord injury via ROS production, tissue acidification or autophagy mechanisms [41–43]. However, it is still unknown whether Hv1 is functional in spinal cord microglia that is important for neuropathic pain development. To this end, we examined the neuropathic pain behaviors, glial activation, and cytokine profile after peripheral nerve injury using Hv1 knockout (KO) mice. We used male mice in the current study due to the critical sex differences in microglial involvement of neuropathic pain [44].

## Results

### The Hv1 proton channel is functionally expressed in spinal microglia and is upregulated after peripheral nerve injury

**Fig. 1:**
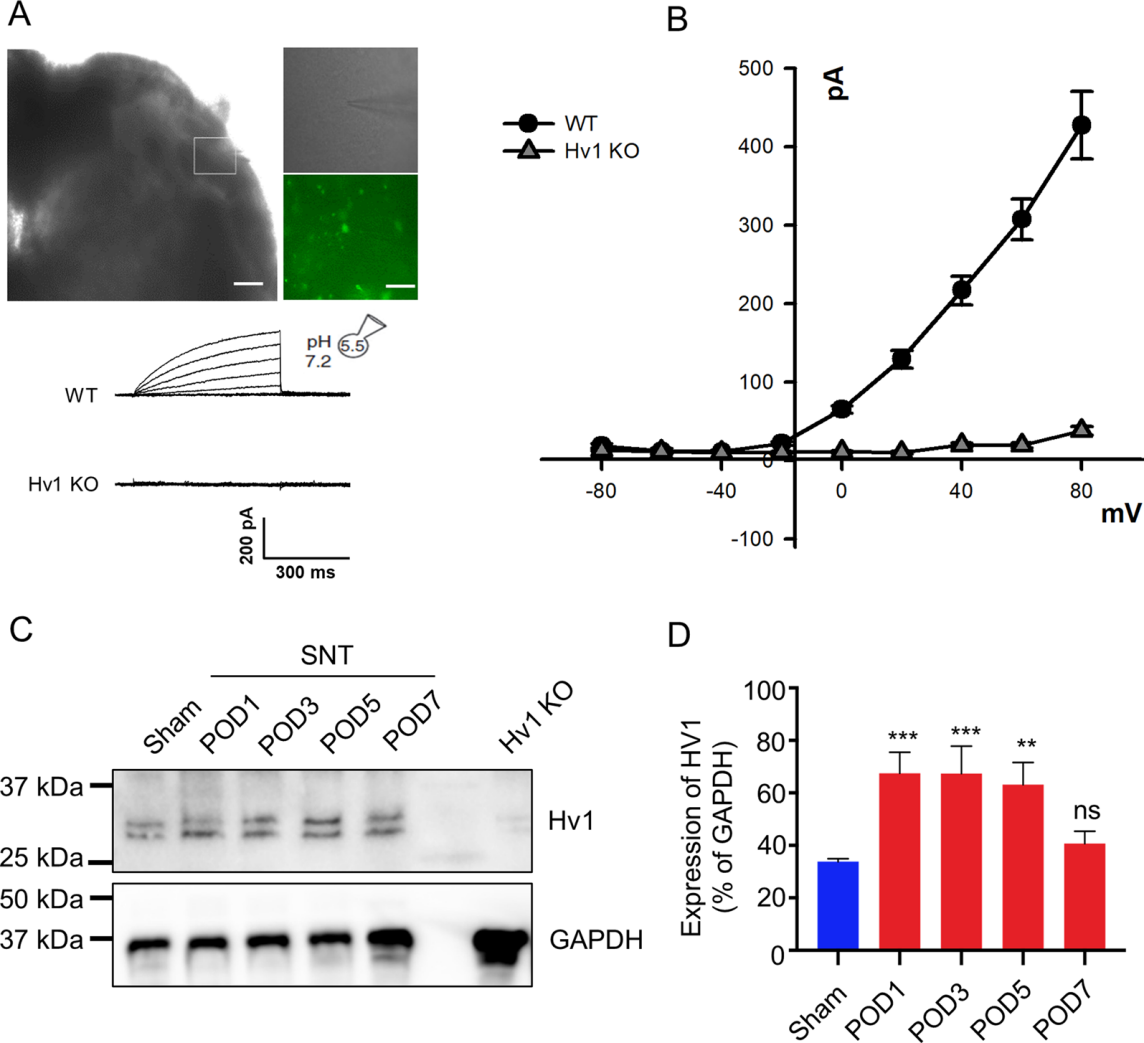
Hv1 is functionally expressed in spinal microglia and upregulated after SNT. **A** Representative images of an acute spinal cord slice with a patched GFP-expressing microglia (boxed area was enlarged, upper). Scale bar: 200 µm (left), 40 µm (right). Outward proton currents induced by voltage steps in spinal cord microglia (lower). Increased pH_i_ decreased the outward proton current and shifted the activation threshold to more depolarized potentials in WT microglia. No outward current was observed in Hv1 KO microglia. **B** I/V curves showing that Hv1 KO abolished voltage-gated proton currents in spinal cord microglia (n = 6 cells from 3 mice each group). **C, D** Representative Western blot images (**C**) and quantification data (**D**) showing that Hv1 expression in the L4-5 level of the dorsal horn increased at POD1-7 after SNT. Data are presented as mean ± SEM, n = 4–5 mice/group. **p < 0.01, ***p < 0.001, GAPDH was used as internal control. Spinal cord tissue from Hv1 KO animals was used as a negative control to confirm the antibody specificity, one-way ANOVA with multi comparisons

Gene profiling in the spinal cord after peripheral nerve injury showed the regulation of numerous genes related to immune function [45, 46]. In addition, activated microglia undergo proliferation in the ipsilateral dorsal horn of the spinal cord after peripheral nerve injury and promote neuropathic pain [12, 47, 48]. Hv1 proton channel is a major ion channel in microglia. However, the function of Hv1 in spinal cord microglia in pain is largely unknown. Using CX_3_CR1^GFP/+^ mice, we performed whole-cell recording in GFP-labeled microglia from acute spinal cord slices harvested from naïve mice (Fig. 1A). At a holding potential of − 60 mV with intracellular pH5.5, a series of depolarizing voltage steps induced outward proton currents in spinal microglia in WT mice. The voltage-gated proton currents were likely mediated by the Hv1 channel, as currents were totally abolished in Hv1 KO mice (Fig. 1A, B). Therefore, the Hv1 channel is functionally expressed in spinal microglia as like that in brain microglia [32].

To examine if Hv1 protein expression is regulated after peripheral nerve injury, we used spinal nerve transection (SNT) in mice, a well-established mouse model of neuropathic pain. Spinal dorsal horn tissues were collected for Western blot analysis following SNT at various postoperative days (POD). We found that the Hv1 protein levels were significantly upregulated at POD1 to POD5 (Fig. 1C, D). The transient upregulation of spinal Hv1 after SNT is paralleled with the critical time window, during which microglia participate in neuropathic pain development [3, 49].

### The Hv1 proton channel contributes to neuropathic pain behaviors after SNT

**Fig. 2:**
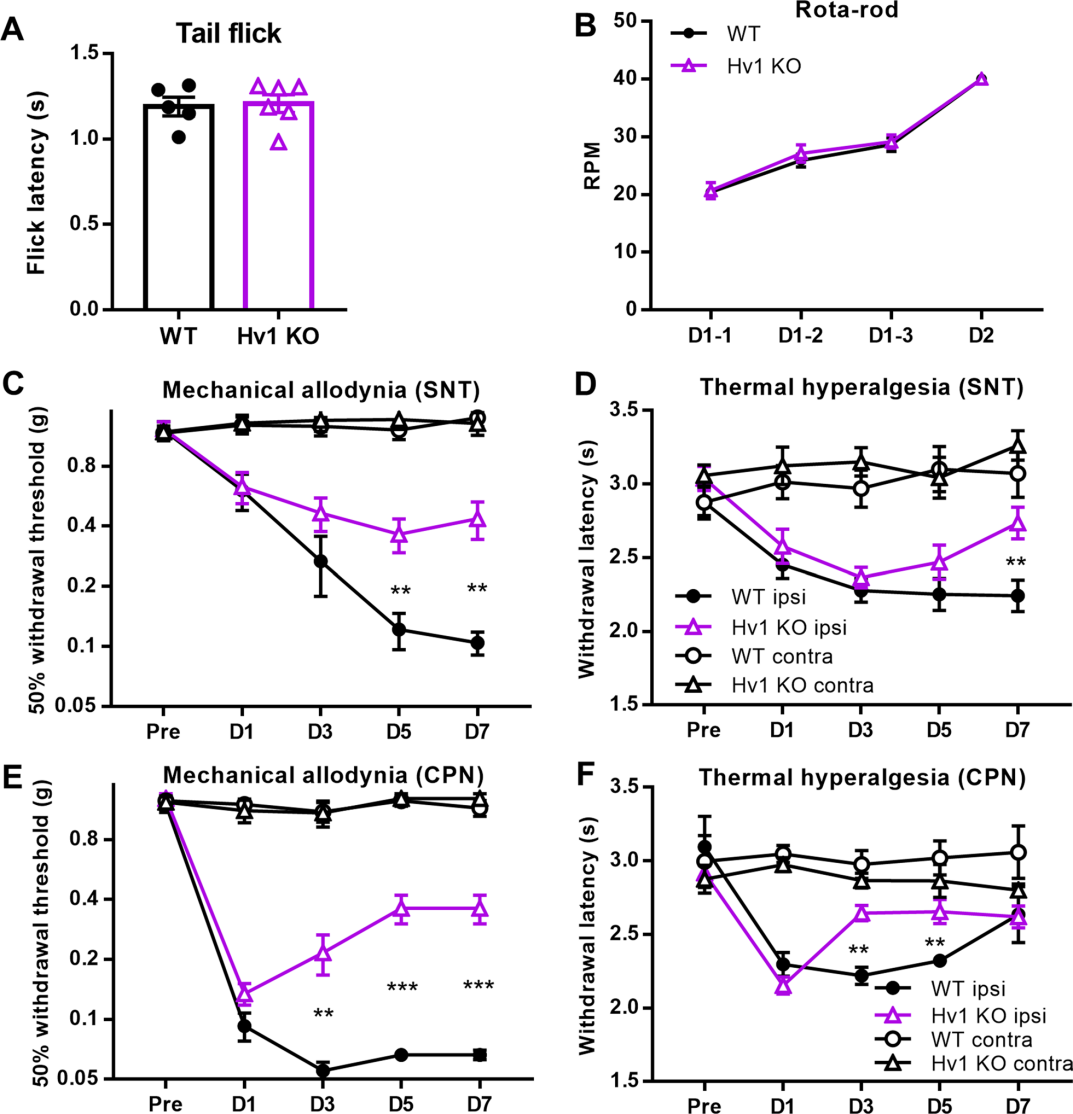
Hv1 is required for pain hypersensitivity after SNT. **A, B** WT and Hv1 KO animals exhibit similar acute pain phenotypes in a tail flick test (A) and motor learning capabilities in a rotarod test (**B**). Data are presented as mean ± SEM, n = 5—6 for each group. WT vs. Hv1 KO., unpaired t-test for tail flick, one-way ANOVA for rotarod. **C, D** Hv1 KO mice display reduced mechanical allodynia (**C**) and thermal hyperalgesia (D) following SNT. Data are presented as mean ± SEM, n = 14 mice for each group. **p < 0.01. WT ipsi vs. Hv1 KO ipsi., unpaired t-test. **E, F** Hv1 KO animals show reduced mechanical allodynia (**E**) and thermal hyperalgesia (**F**) following CPN ligation. Data are presented as mean ± SEM, n = 8 for WT, n = 7 for Hv1 KO. **p < 0.01; ***p < 0.001. WT ipsi vs. Hv1 KO ipsi., unpaired t-test

The upregulation of Hv1 in microglia after SNT suggests that the channel may participate in neuropathic pain. To directly test this idea, we compared the pain behaviors between WT and Hv1 KO mice after SNT. First, acute pain behaviors were tested and we found that Hv1 KO mice exhibited similar responses to a tail flick test as WT mice (Fig. 2A). Moreover, basal motor behaviors in Hv1 WT and KO mice were similar (Fig. 2B). Next, we examined chronic pain behaviors, including mechanical allodynia and thermal hyperalgesia, after SNT. WT mice developed both mechanical allodynia and thermal hyperalgesia that reached a plateau by the third postoperative day (Fig. 2C, D). Hv1 KO mice also developed SNT-induced pain hypersensitivity (Fig. 2C, D). However, Hv1 KO mice showed significant improvement in pain hypersensitivities on POD 5–7 but not POD 1–3 compared with WT mice. The attenuation of neuropathic pain in the later phase following SNT in Hv1 KO mice suggests a possible role of Hv1 channel function in neuropathic pain maintenance.

To confirm that the behavioral phenotype in Hv1 KO mice after SNT extends to other nerve injury models, we repeated the behavioral experiments after ligation of common peroneal nerve ligation (CPN). This neuropathic pain model employs a less invasive procedure and produces little muscle damage. Thus the animals recover quickly from the surgery with less motor dysfunctions [50]. As with the SNT model, the behavioral phenotypes of Hv1 KO mice could be distinguished from WT mice in the CPN model (Fig. 2E, F). Taken together, these results indicate that microglial Hv1 channel plays a significant role in the maintenance of pain hypersensitivities, as demonstrated by two neuropathic pain models.

### Microglial proliferation and MAKP p38 activation in the spinal cord persist in Hv1 KO mice after SNT

**Fig. 3:**
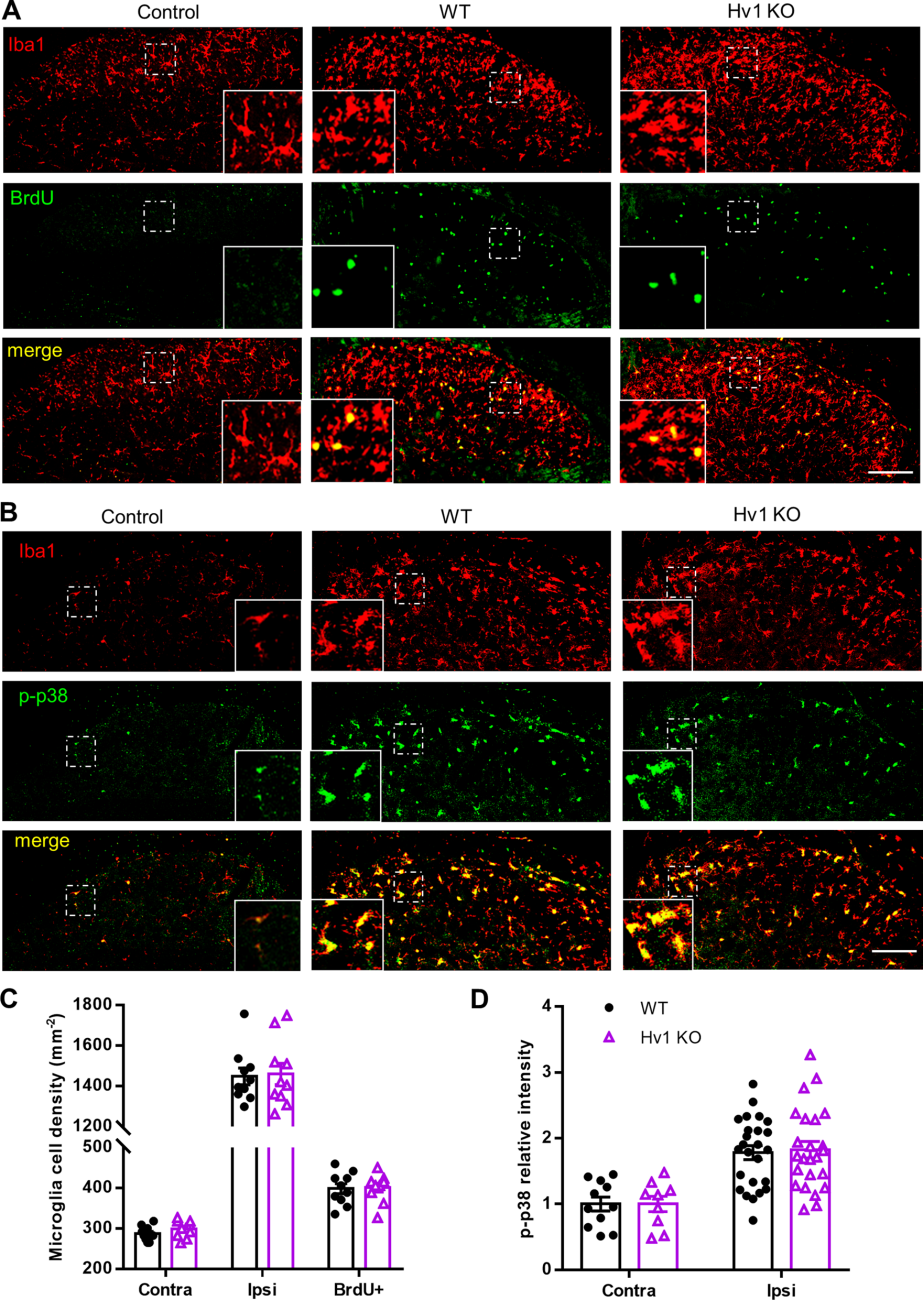
Hv1 deficiency does not alter microglial activation after SNT. **A** Representative images of Iba1 (top panels), BrdU (middle panels) and merged (bottom panels) immunoreactivity in the ipsilateral dorsal horn in control (left), SNT-induced WT (center) and SNT-induced Hv1 KO (right) mice at POD7. Inserts are higher magnification of boxed regions in the representative low magnification images. **B** Representative images of Iba1 (top panels), phosphorylated p38 (p-p38, middle panels) and merged (bottom panels) immunoreactivity in the ipsilateral dorsal horn in control (left), SNT-induced WT (center) and SNT-induced Hv1 KO (right) mice at POD3. Inserts are higher magnification of boxed regions. Scale bar:100 µm. **C** Quantitative summary of microglial density in the contralateral and ipsilateral dorsal horn as well as BrdU + microglia density in the ipsilateral dorsal horn at POD7 following SNT in WT and Hv1 KO mice (n = 10 slices from 3 mice for each group, unpaired t-test). **D** Quantitative p-p38 immunoreactivity in the contralateral and ipsilateral dorsal horn of WT and Hv1 KO mice. Data are presented as mean ± SEM, cells were randomly picked from 6 slices for each group. WT vs. Hv1 KO., unpaired t-test

Robust proliferation of spinal microglia occurs after SNT and is an important feature of microgliosis during neuropathic pain [12, 47]. Since the Hv1 channel is specifically expressed in microglia, we first examined microgliosis after SNT beginning with microglial proliferation. By immunostaining of microglial marker Iba1 in mice, we quantified microglial cell numbers in the ipsilateral dorsal horn at POD7 after SNT. For proliferation, we labeled spinal cord tissues with 5-bromo-2′-deoxyuridine (BrdU) (Fig. 3A). Our results showed that microglial cells in the ipsilateral dorsal horn were about 5 folds that of the contralateral dorsal horn for both WT and Hv1 KO mice. The density of BrdU-positive microglia was about 30% and also similar between WT and Hv1 KO mice (Fig. 3C). These data indicate that SNT-induced microglial cell proliferation was not affected by Hv1 deficiency.

MAPK p38 is predominantly expressed in spinal microglia and p38 phosphorylation (p-p38) is critical for microglial activation, which triggers several downstream pathways such as P2X4R upregulation, BDNF/cytokine release, and proliferation following neuropathic pain [51, 52]. Thus, we examined the p-p38 expression using double immunostaining together with Iba1 at POD3 after SNT. We found that p-p38 positive signals expressed in Iba1^+^ microglia (Fig. 3B). In the ipsilateral dorsal horn, Iba1-labelled microglia showed a hypertrophic morphology with enlarged somata compared to those in the contralateral dorsal horn (see inserts in Fig. 3B). Statistical analysis showed that the fluorescent intensity of p-p38 signals was significantly increased in the ipsilateral dorsal horn compared to the contralateral side and there was no difference between WT and Hv1 KO mice (Fig. 3D). Together, our results indicate that Hv1 deficiency did not affect spinal microglial activation as determined by microglial cell numbers, proliferation, and p38 activation following SNT.

### Hv1-dependent ROS production in microglia contributes to the neuropathic pain

**Fig. 4:**
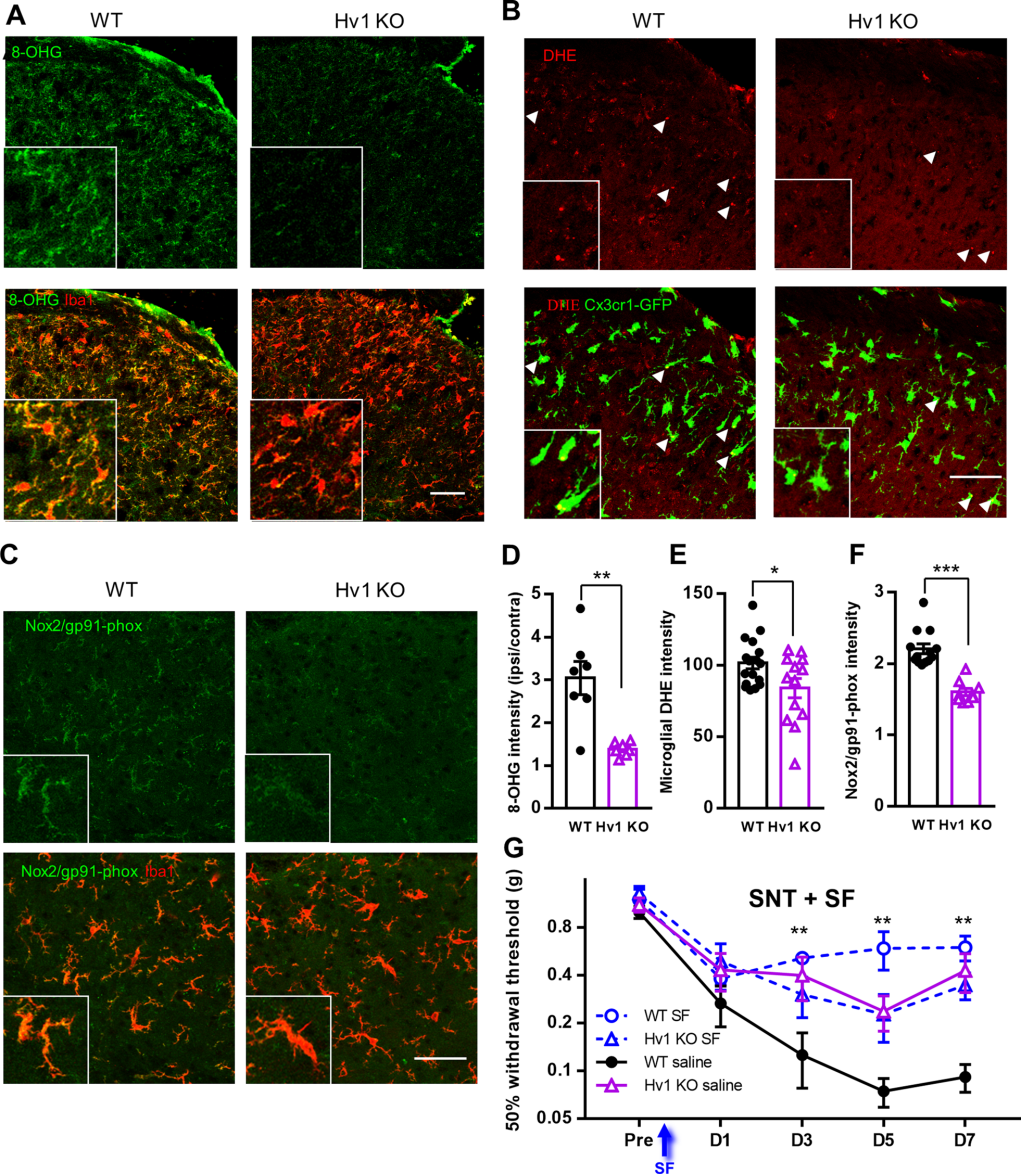
SNT-induced ROS production is attenuated in Hv1 KO mice. **A** Representative images of 8-OHG (top) and merged 8-OHG/Iba1 (bottom) immunostaining in WT (left) and Hv1 KO (right) spinal cord at POD7 after SNT. **B** Representative images of ox-DHE (top) and merged ox-DHE/CX_3_CR1-GFP (bottom) signals in WT (left) and Hv1 KO (right) spinal cord at POD3 after SNT. **C** Representative images of Gp91^phox^ (top) and merged Gp91^phox^/Iba1 (bottom) immunostaining in WT (left) and Hv1KO (right) spinal cord at POD1 after SNT. Scale bar: 50 µm. **D–F** Quantitative data showing 8-OHG, ox-DHE and Gp91^phox^ signal in the ipsilateral dorsal horn after SNT. Data are presented as mean ± SEM, 7–15 slices from 3 mice for each group. *p < 0.05; **p < 0.01; ***p < 0.001. WT vs. Hv1 KO unpaired t-test. **G** Mechanical allodynia in WT and Hv1 KO mice treated with and without ROS scavenger, sulforaphane (SF), 1 h after SNT (arrow). SF treatment significantly attenuated mechanical allodynia in WT mice when compared to control group (WT + saline) but not in H1KO mice with SF or saline treatment. Data are presented as mean ± SEM, n = 7 mice for each group. **p < 0.01; ***p < 0.001, unpaired t-test

Hv1 channel is known to participate in NOX-dependent ROS production in leukocytes and microglia [32, 53]. The proton current through Hv1 provides charge compensation and balances intracellular acidification during ROS production [54, 55]. Increased and oxidized nucleic acid by ROS can be detected using an 8-hydroxyguanine (8-OHG) antibody in the spinal cord dorsal horn [22]. We found indeed 8-OHG signals were increased in Iba1-positive microglia from ipsilateral dorsal horn after SNT. However, the 8-OHG signal was largely abolished in Hv1 KO mice (Fig. 4A). The overall 8-OHG intensity in the ipsilateral horn was 3 folds that of the contralateral horn in WT mice and 1.4 fold that of the Hv1 KO ipsilateral horn (Fig. 4D). The overall background intensity of the contralateral horn was not different between WT and Hv1 KO samples (data not shown). Next, we measured superoxide generation in L4 microglia with oxidized-Dihydroethidium (ox-DHE) after SNT. DHE (10 µg in 5 µl ACSF) was intrathecally injected 2 h before the mice were sacrificed. Positive ox-DHE signals appear as bright red puncta within microglia (Fig. 4B). Consistent with the results using 8-OHG, the specific ox-DHE signal on microglia from Hv1 KO samples was significantly weaker than that from the WT (Fig. 4E). Finally, we also investigated the functional expression of NADPH oxidase (NOX) in Hv1 KO mice following SNT by immunostaining for gp91^phox^ (NOX2). We found Hv1 KO spinal cords exhibited significantly lower gp91^phox^ expression compared to Hv1 WT tissues 3 days after SNT (Fig. 4C, F). Therefore, our results demonstrate that ROS production was impaired in Hv1 KO mice after peripheral nerve injury.

Considering the important role of ROS in the chronic pain pathogenesis, the abrogation of ROS production in Hv1 KO mice may contribute to the attenuation of pain hypersensitivities after SNT. To test this idea, ROS scavenger sulforaphane (SF, 50 mg/kg, i.p.), which exerts its antioxidant effect by inducing nuclear translocation of Nrf-2 with subsequent heme oxygenase-1 (HO-1) expression in myeloid cells [22], was treated at 1 h following SNT. We found that treatment of WT mice with SF attenuated mechanical allodynia (Fig. 4G). However, SF did not affect pain hypersensitivities of Hv1 KO mice after SNT (Fig. 4G). Together, these results indicate that Hv1 deficient animals generate less ROS, which contributes to the amelioration of neuropathic pain phenotypes after SNT.

### Astrocyte activation was reduced in the spinal cord of Hv1 KO mice after SNT

**Fig. 5:**
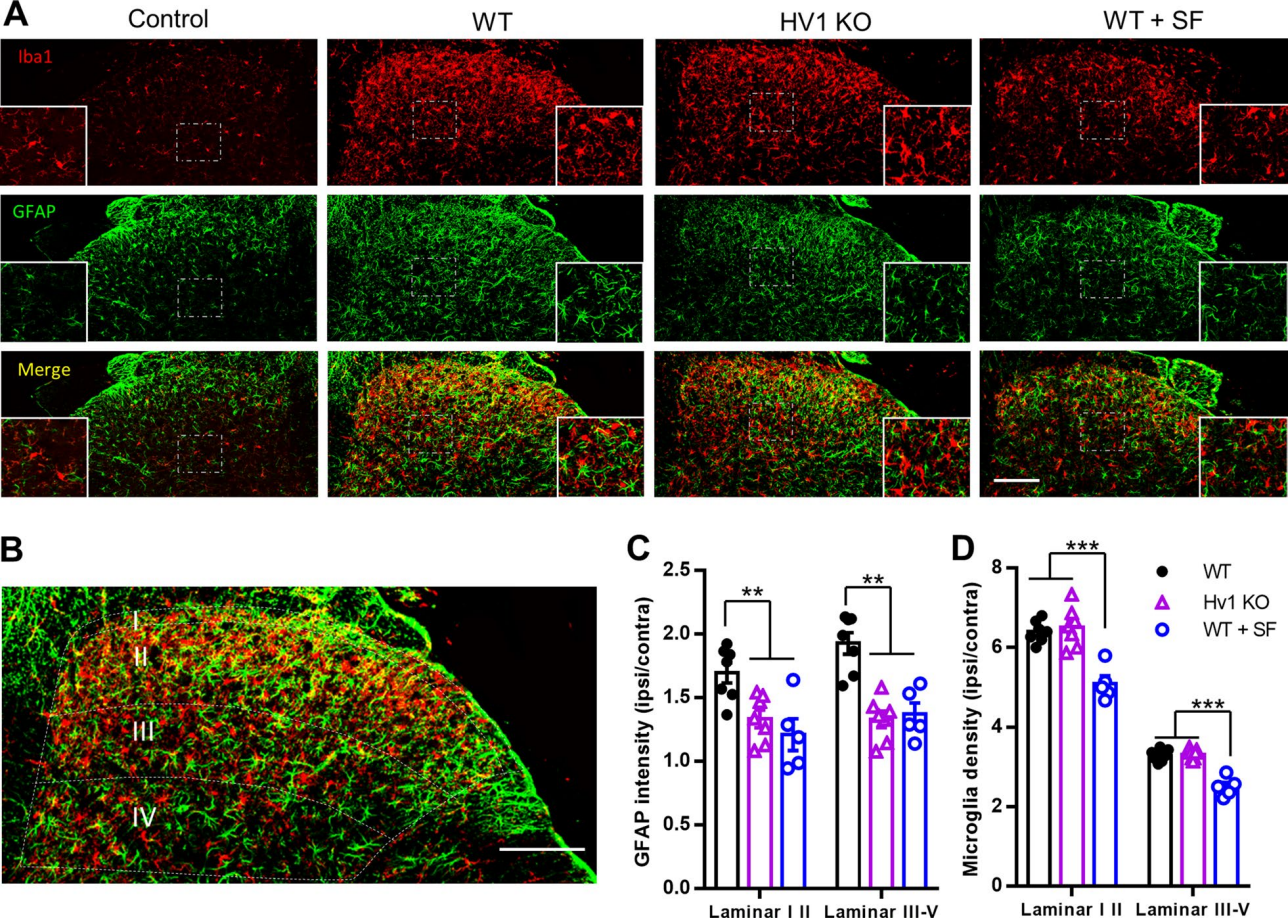
Reduced astrocyte activation in Hv1 KO spinal cord after SNT. **A** Representative images of Iba1 (top panels), GFAP (middle panels) and merged (bottom panels) immunoreactivity in dorsal horn of control (the contra side of a WT sample was shown, left), the ipsilateral of SNT-induced WT (left center), SNT-induced Hv1 KO (right center), and sulforaphane-treated SNT-induced WT (right) mice at POD 7. Inserts are higher magnification of boxed regions. Scale bar: 100 µm. **B–D** Sample dorsal horn slice (**B**) is labelled with GFAP-astrocytes (green) and Iba1-microglia (red) highlighting the different lamina of the spinal cord. Astrocytes (**C**) and microglia (**D**) density were quantified in WT and Hv1 KO tissues at POD 7. Scale bar: 100 µm. Data are presented as mean ± SEM, images obtained from 3 mice for each group. **p < 0.01; ***p < 0.001, unpaired t-test

Astrocytes are important for neuropathic pain development, especially in the late maintenance phase [24, 56]. The fact that the neuropathic pain attenuation in Hv1 KO mice appeared in the late phase led us to study astrocyte involvement. SNT induced a dramatic GFAP expression on the ipsilateral dorsal horn on POD7 compared to contralateral side, which appeared in both superficial and deeper lamina in WT mice (Fig. 5A, C). In Hv1 KO mice, GFAP induction was significantly reduced (Fig. 5A, C). To test the role of Hv1-dependent ROS production in astrocyte activation, we examined the effect of SF treatment. We found that GFAP expression in WT mice was greatly reduced in both superficial and deep lamina after SF treatment (Fig. 5A, C). Notably, SF treatment also reduced microglial activation shown as reduced cell densities compared with saline group (Fig. 5D). These results strongly indicate that SNT induced spinal astrocyte activation requires Hv1 and ROS production in microglia.

### Reduced astrocytic IFN-γ mediates attenuated pain hypersensitivity in Hv1 KO mice after SNT

**Fig. 6:**
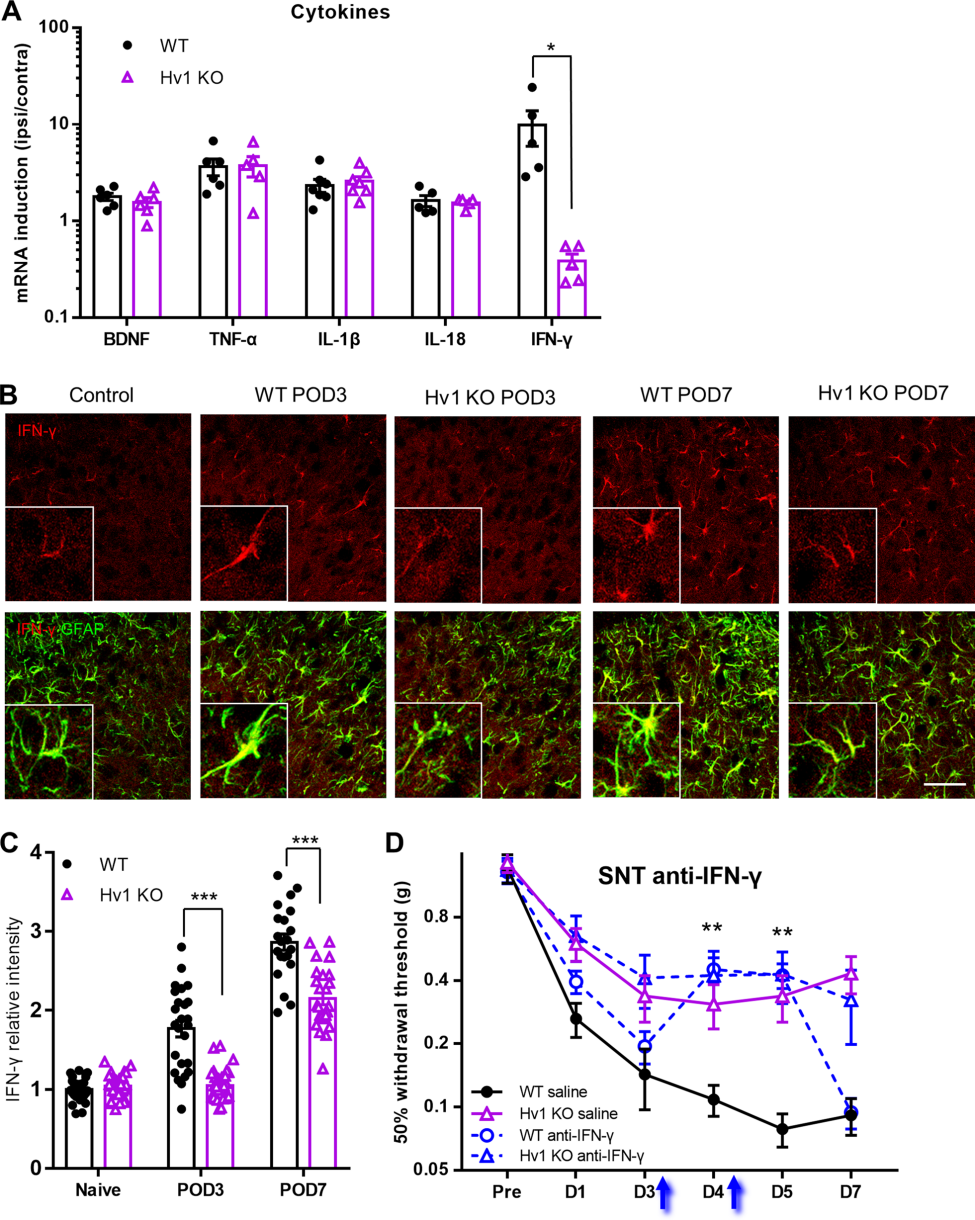
Hv1 is required for astrocyte-derived IFN-γ to promote neuropathic pain after SNT. **A** Real-time PCR analysis data showing mRNA expression fold change of glial-derived cytokines in the spinal cord at POD3 after SNT. Data are presented as mean ± SEM, n = 6 runs from 3 sample pools, each sample pool contained 2 mouse samples for each group**.** *p < 0.05 unpaired t-test. **B** Representative images of IFN-γ (top panels) and IFN-γ / GFAP merged (bottom panels) immunoreactivity in the ipsilateral dorsal horn in control (a naïve WT sample was shown, left), POD3 SNT-induced WT (left center), and Hv1 KO (center), as well as POD7 SNT-induced WT (right center) and Hv1 KO (right) tissues. Inserts are higher magnification of boxed regions. Scale bar: 50 µm. **C** Quantitation of IFN-γ immunoreactivity in control as well as POD3 and 7 SNT-treated WT and Hv1 KO mice. Data are presented as mean ± SEM, cells were randomly picked from 3 mice for each group. ***p < 0.001. WT vs. Hv1 KO, unpaired t-test. **D** Mechanical allodynia in WT and Hv1 KO mice treated with or without neutralizing IFN-γ antibody (anti-IFN-γ). Anti-IFN-γ (1 μg in 5 μl ACSF) was applied intrathecally at POD3 and POD4 after SNT. Anti-IFN-γ intrathecal injection attenuated mechanical allodynia in WT mice when compared to the control group (WT + saline) but not in HV1 KO mice with anti-IFN-γ or saline injection. Data are presented as mean ± SEM. n = 7 mice for each group, **p < 0.01, unpaired t-test

Since spinal cytokines are critical for neuropathic pain pathogenesis [3, 5, 57], we investigated potential differences between cytokine expression in WT and Hv1 KO mice. The mRNA expression levels were examined using real-time PCR from spinal cord L4 segments at POD3 after SNT. Interestingly, we found that BDNF, TNF-α, IL-1β, and IL-18 were expressed at similar levels in WT and Hv1 KO tissues. However, IFN-γ expression levels were abolished in Hv1 KO tissues (Fig. 6A).

IFN-γ is reported to be able to activate microglia through IFN-γ receptors and induce chronic pain [23, 45]. The absence of IFN-γ induction we observed in Hv1 KO mice after SNT could contribute to neuropathic pain attenuation. To test this hypothesis, we first used fluorescent immunostaining to detect the SNT induced IFN-γ expression in spinal dorsal horn on both POD3 and POD7 (Fig. 6B). We found that the intensities of IFN-γ were positively correlated with hypertrophic astrocyte morphologies and high expression of GFAP (Fig. 6B, inserts). The basal expression of IFN-γ in the contralateral dorsal horn of Hv1 KO mice was similar to that in WT tissues. However, IFN-γ induction in the ipsilateral dorsal horn was largely abolished at POD3 and POD7 after SNT (Fig. 6C).

Next, to directly test the function of IFN-γ in the spinal cord, we intrathecally injected IFN-γ neutralizing antibodies (1 μg in 5 μl ACSF) into WT mice after neuropathic pain was established following SNT. We found that neutralizing endogenous IFN-γ partially reversed the mechanical allodynia within 24 h of the infusion of the antibody. However, the mechanical allodynia returned to saline group level after 72 h (Fig. 6D). In the Hv1 KO mice, IFN-γ antibody treatment after SNT did not affect the behavioral responses at any time point. These results suggest that endogenous IFN-γ and Hv1 are both necessary for neuropathic pain maintenance.

### Microglial Hv1 and astrocytic IFN-γ mediate microglia-astrocyte interaction in pain hypersensitivities

**Fig. 7:**
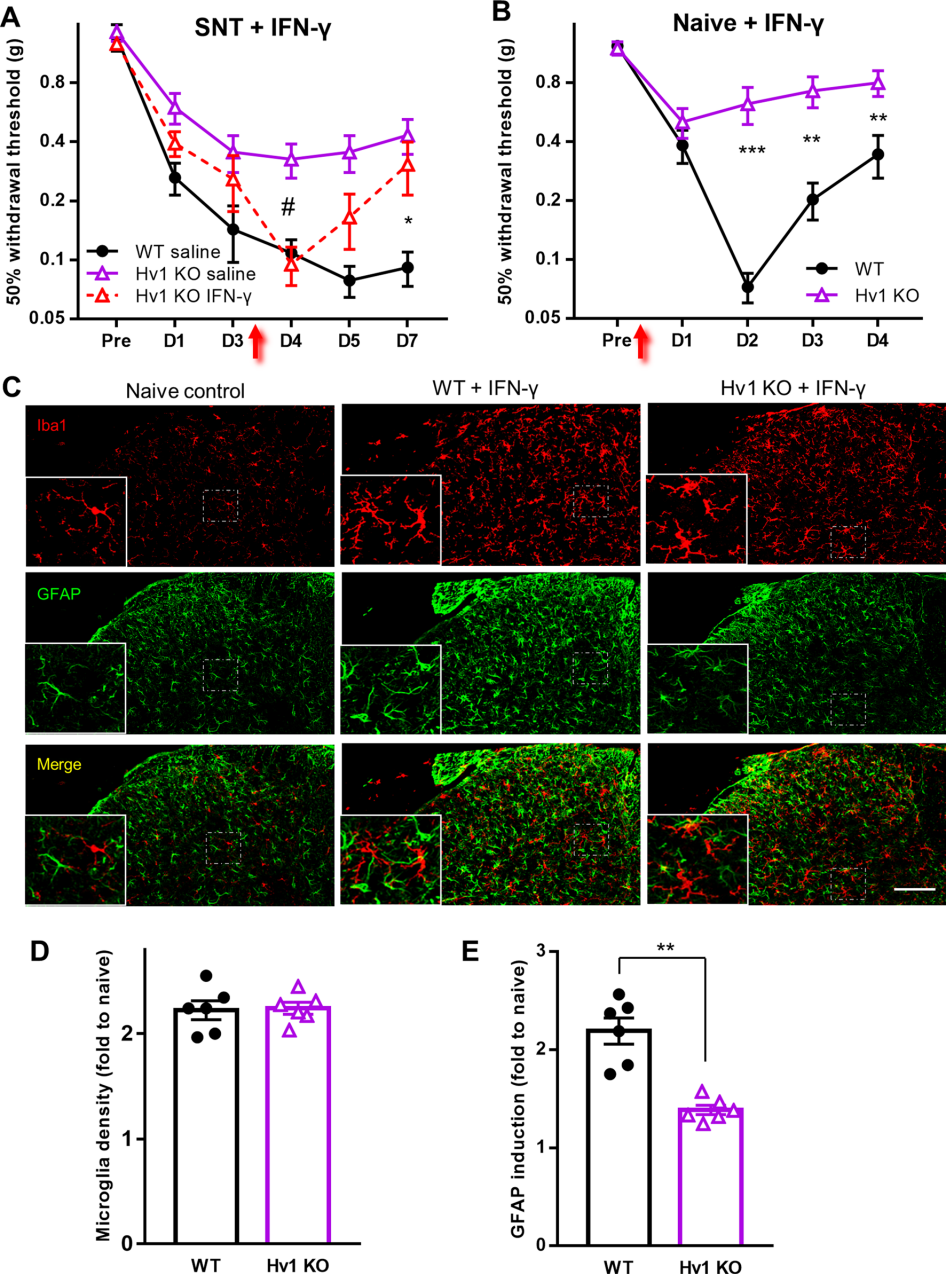
Hv1 is required for IFN-γ promotes neuropathic pain and astrocyte activation. **A** Mechanical allodynia in SNT mice treated with exogenous IFN-γ. IFN-γ (100U in 5 μl ACSF) was intrathecally applied at POD3 after SNT. Data are presented as mean ± SEM, n = 7 for control groups, n = 6 for IFN-γ treated group. ^#^*p* < 0.05, Hv1 KO + saline vs Hv1 KO + IFN-γ, *p < 0.05, WT + saline vs. Hv1 KO + IFN-γ, unpaired t-test. **B** Mechanical allodynia in naïve mice treated with exogenous IFN-γ. IFN-γ (100U in 5 μl ACSF) was intrathecally applied after the baseline pain test. Data are presented as mean ± SEM, n = 6 mice for each group. **p < 0.01, ***p < 0.001. WT vs. Hv1 KO, unpaired t-test. **C** Representative images of Iba1 (top panels), GFAP (middle panels) and merged (bottom panels) immunoreactivity in the dorsal horn in control (a naïve WT sample was shown, left), IFN-γ–treated WT (center) IFN-γ–treated Hv1 KO (right) mice at day 4 post injection. Scale bar: 50 µm. **D**, **E**, Quantification of microglia (**D**) and astrocyte (**E**) density at POD4 in Hv1 WT and KO mice following IFN-γ treatment. Data are presented as mean ± SEM, n = 6 slices from 3 mice for each group. **p < 0.01. WT vs. Hv1 KO, unpaired t-test

The reduced astrocytic IFN-γ expression and abolished IFN-γ-dependent neuropathic pain in Hv1 KO mice indicate microglial Hv1 and astrocytic IFN-γ mediates the microglia-astrocyte interaction underlying pain hypersensitivities. To test this hypothesis, we intrathecally injected IFN-γ (100U in 5 μl ACSF) at POD3 after SNT and then examined the pain hypersensitivities in Hv1 KO mice. We found that IFN-γ could restore the mechanical allodynia in Hv1 KO mice, decreasing the paw withdrawal threshold to the WT mice levels at 24 h after SNT (Fig. 7A). The rescue effect was transient and the pain hypersensitivities gradually disappeared at POD 5 and 7 (Fig. 7A).

Exogenous IFN-γ triggers microglial proliferation and increases pain hypersensitivities [23]. To clarify the Hv1 role in IFN-γ-induced pain increase, we performed IFN-γ infusion experiments with both naïve WT and Hv1 KO mice by intrathecal injection of IFN-γ (100U in 5 μl ACSF). Mechanical allodynia was successfully induced in WT mice with a peak at 2 days after the injection. In Hv1 KO mice, a similar lower threshold decrease appeared at 24 h after the injection but then recovered quickly on subsequent days (Fig. 7B). Iba1 staining showed that microglia cell densities were doubled compared with the naive mice in both the WT and Hv1 KO groups (Fig. 7C, D). Thus, exogenous IFN-γ can induce proliferation of Hv1 KO microglia similarto WT ones. To study if astrocytes were activated subsequently to IFN-γ infusion, GFAP immunostaining was performed on IFN-γ-infused animals. We found that IFN-γ treatment induced robust astrocyte activation (GFAP expression) in WT mice, while GFAP upregulation was much less pronounced in the Hv1 KO mice (Fig. 7C, E). Therefore, these results suggest that IFN-γ induced allodynia requires Hv1-dependent astrocyte activation.

## Discussion

**Fig. 8:**
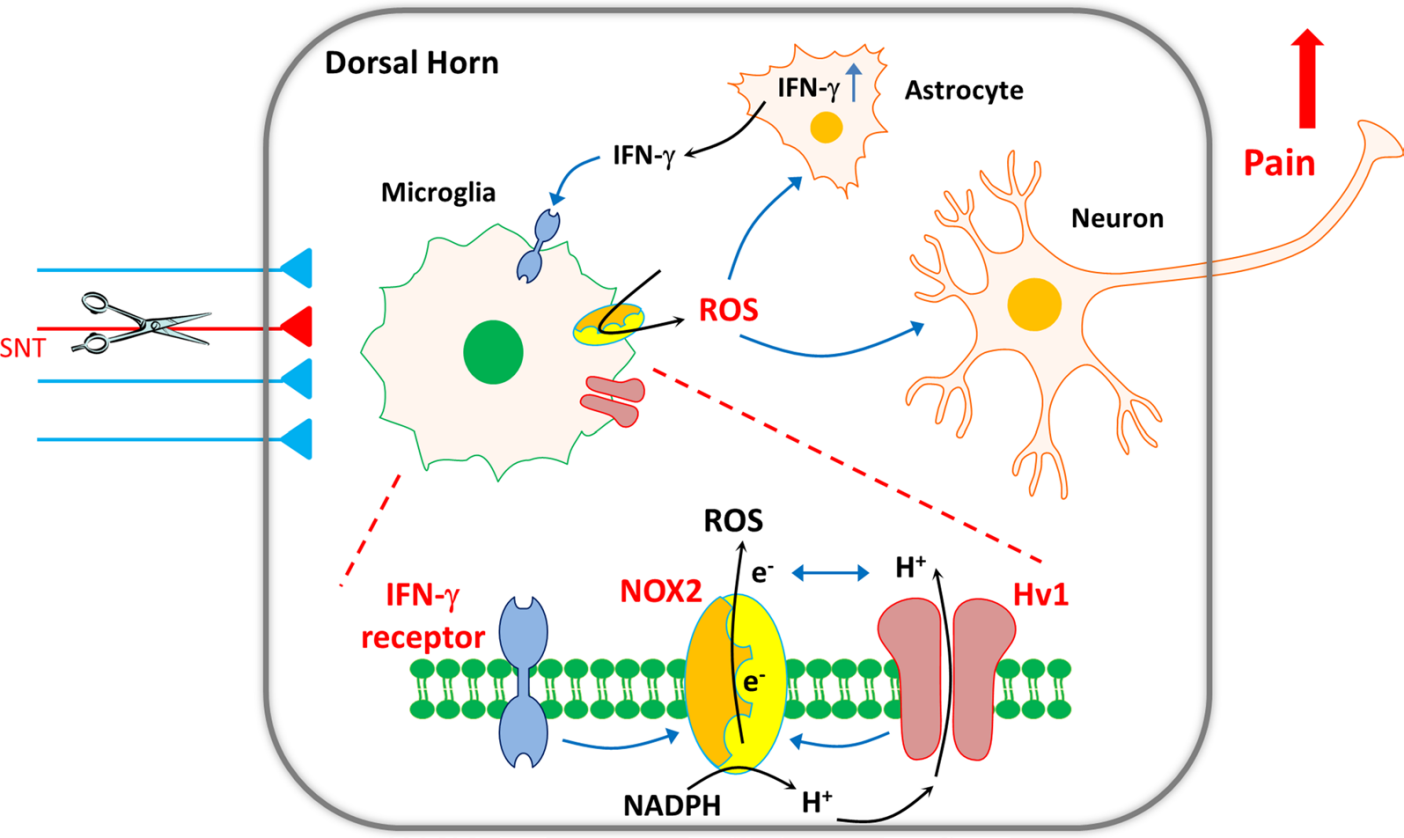
Schematic diagram showing Hv1-dependent microglia-astrocyte interaction in neuropathic pain. After peripheral nerve injury, spinal microglia are activated and accompanied with Hv1 proton channel dependent ROS production. ROS acts on both neurons and astrocytes, triggering IFN-γ release. IFN-γ in turn contributes to further microglia activation and microglia-astrocyte interaction to maintain pain hypersensitivity after SNT

The current study demonstrates that microglial Hv1 proton channels contributed to ROS generation, astrocyte activation, IFN-γ production, and the subsequent neuropathic pain following peripheral nerve injury (Fig. 8). These results provide novel insights into Hv1 function in microglia-astrocyte interaction and the pathogenesis of neuropathic pain. The microglial Hv1-astrocytic IFN-γ axis of communication has not been previously appreciated. Particularly, this study provides molecular mechanisms underlying microglial Hv1 in microglia-astrocyte communication and a rationale for microglial Hv1 as a novel therapeutic target for the pain management.

### Hv1 channel of spinal microglia in ROS production in neuropathic pain

ROS can be generated by NOX activation via transferring electrons from NADPH across the membrane and coupling these to molecular oxygen [58, 59]. The electron transfer rapidly depolarizes and acidifies these cells. Given that excessive depolarization and intracellular acidification inhibit further expulsion of electrons, a charge-compensating mechanism is needed to maintain NOX activity. Current evidence indicates that Hv1 is activated by NOX-dependent acidosis and membrane depolarization, as well as required for NOX-dependent ROS production in a variety of cells, including neutrophils [33], B cells [34], and eosinophils [35]. Our previous work indicated that functional Hv1 is exclusively expressed in microglia in the CNS, and was critical for NOX-dependent ROS generation in microglia in situ and in vitro [32, 36, 37, 42]. In the current study, we extend the function of microglial Hv1 into the spinal cord during neuropathic pain (Figs. 1, 2).

In the spinal cord, microglial NOX2 contributes to most of the SNT-induced ROS production as both microglial activation and neuropathic pain initiation were reduced in NOX2 KO mice [22]. However, in our study, microglial activation was not altered and neuropathic pain attenuation appeared in the maintenance phase in the Hv1 KO mice (Fig. 2), suggesting that the efficiency of ROS elimination is different between NOX2 and Hv1 KO mice. Although we found a dramatic reduction in ROS-induced nucleic acid oxidization as indicated by 8-OHG staining in Hv1 KO mice, ROS production was still detectable within microglia as indicated by DHE staining (Fig. 4). These results suggest that the NOX activation was successfully initiated, but the ROS production could not persist after intracellular acidification as well as NOX2 upregulation was inhibited in Hv1 KO mice. In addition, NOX2 is partially functional in Hv1 KO microglia after SNT, which might be sufficient to trigger microglial activation. Alternatively, mediators responsible for SNT-induced microglial activation could be released independently of Hv1 function. Considering the important function of dorsal root ganglion (DRG) macrophages in neuropathic pain [60], whether the Hv1 channel in DRG macrophages contributes to pain phenotype in general KO mice warrants the further investigation.

### Hv1-dependent microglia-astrocyte communication in neuropathic pain

Progress in the RNA sequencing (RNA-seq) techniques demonstrated a multiple spectrum of reactive microglial subtypes in the diseased conditions [61, 62]. Recent RNAseq studies showed that pro-inflammatory cytokine and immunomodulatory drivers: complements, FcɣRs, and INFs are the important upstream mediators of nerve injury-induced gene regulation in reactive microglia in abnormal maladaptive state over long periods of pain hypersensitivity [16]. It was well documented that microglia are critical in developing neuropathic pain especially in the initiation, during which, the release of BDNF, cytokines such as TNF-α and IL-1β are essential for the establishment of pain hypersensitivities [63, 64]. Here, we show that SNT-induced mRNA expression of these molecules was maintained at POD3 in Hv1 KO mice (Fig. 6A), which is consistent with the observation of the normal initiation of neuropathic pain. Microglial activation reaches peak levels at around 3 days after nerve injury as indicated by proliferation rates and p-38 activation [12, 51, 65]. Our previous study found that microglia ablation before SNT could delay neuropathic pain, while microglia ablation at day 7 after SNT had very limited effects [64], consistent with several pharmacological studies with minocycline [66]. On the other hand, fluorocitrate, an inhibitor of astrocyte function, could reverse mechanical allodynia during the maintenance phase of neuropathic pain [66–68]. The behavioral phenotype seen in Hv1 KO mice in which pain hypersensitivities were attenuated in the maintenance phase led us to consider a possible role of microglia-astrocyte communication. Indeed, we observed reduced astrocyte activation as indicated by GFAP expression on POD7 in Hv1 KO mice (Fig. 5). Moreover, ROS scavenger sulforaphane dramatically inhibited SNT induced GFAP expression in WT mice. These results indicate the function of Hv1-dependent ROS in astrocytic activation and microglia-astrocyte interaction during SNT-induced neuropathic pain.

Recent studies have suggested intriguing interaction between microglia and astrocyte in a variety of neurological disorders [69–71]. Complement signaling and interleukins were shown to be the potential mediators for microglia-astrocyte interaction. This A1 type of astrocyte activation was also confirmed in a chronic post-surgical pain model in the spinal cord [72]. Here we demonstrate Hv1-dependent ROS production in microglia-astrocyte communication that drives neuropathic pain after peripheral nerve injury. In addition to ROS production, recent studies raised an intriguing possibility that Hv1-mediated proton extrusion could be important for microglial Hv1 function in brain injury and spinal cord injury [30, 38, 41]. Hv1-mediated proton release could lead to tissue acidosis, which activates proton-gated ion channels expressed in spinal neurons [73–75]. Future studies are needed to test whether protons released by Hv1 could regulate microglia-astrocyte communication important for the pathogenesis of neuropathic pain.

### Astrocyte activation, IFN-γ production and neuropathic pain

Accumulating evidence has shown the importance of astrocyte activation in neuropathic pain, which includes persistent activation of p-ERK, p-JNK and release of IL-1β, IFN-γ, MCP-1 and CXCL1 [24, 49]. One of the astrocyte activation mechanisms involves IL-18 released by microglia. IL-18 receptors are expressed in astrocyte and their activation are necessary for neuropathic pain development [76]. Here, we show that in Hv1 KO mice, SNT-induced IL-18 mRNA expression was intact but GFAP expression and astrocyte related cytokine (IFN-γ) activation were reduced (Fig. 6A). In addition, the elimination of ROS either in Hv1 KO tissues or by application of a ROS scavenger was correlated with a reduction of GFAP expression (Figs. 4, 5). These results together strongly suggest a role of ROS in promoting spinal astrocyte activation after nerve injury.

In cultured rat brain astrocytes, TGF-β1 activates ROS-dependent signaling pathway [77], including the extracellular signal-regulated kinase 1/2 (ERK1/2), c-Jun-N-terminal kinase (JNK) and NF-κB. In the spinal cord, NOX2 is predominantly expressed in microglia [22]. Thus, microglial-derived ROS production could be involved in the astrocyte ERK and JNK activation to trigger the downstream NF-κB and matrix metalloproteinase (MMP) pathways. Indeed, several lines of evidence showed that MMP9 and MMP2 are involved in the neuropathic pain development during the early initiation and late maintenance phases, respectively [78, 79]. Further study is needed to elucidate the molecular mechanisms by which ROS activates astrocytes in the spinal cord during neuropathic pain.

IFN-γ is able to exert strong pro-inflammatory effects by neuronal and microglial activation [3, 57]. IFN-γ may reduce GluR1 expression on dorsal horn inhibitory neurons and result in the disinhibition of synaptic activity and central sensitization [80]. Here, we showed that neutralizing spinal endogenous IFN-γ partially and transiently reversed pain allodynia (Fig. 6). Injection of IFN-γ at POD3 after SNT could restore the mechanical allodynia in HV KO mice (Fig. 7A). In addition, mechanical allodynia was successfully induced by IFN-γ injection in naïve WT mice (Fig. 7B) indicating that persistent IFN-γ expression is necessary for neuropathic pain maintenance. IFN-γ receptor deficient mice showed attenuated neuropathic pain [23]. Although IFN-γ is released by astrocytes, the IFN-γ receptor is mainly expressed by microglia [23]. In addition, IFN-γ receptor deficiency reduced microglial activation after nerve injury, while intrathecal injection of IFN-γ induced microglial proliferation and pain hypersensitivity that lasts for several days [23]. In the current study, we found IFN-γ was still able to induce microglial activation in Hv1 KO mice, but could not trigger long-lasting pain hypersensitivity. However, the different timeline of Hv1 upregulation (POD1-5) and IFN-γ upregulation (POD3-7) suggests that microglial Hv1 activation and ROS generation could be the trigger for IFN-γ upregulation and astrocyte activation after peripheral nerve injury. Moreover, reactive astrocytes expressing pro-inflammatory cytokine such as IFN-γ could be neurotoxic reactive A1 type in neuropathic pain [72]. Therefore, future study is needed to determine how astrocytic IFN-γ upregulation promotes spinal neuron excitability and the central sensitization to induce neuropathic pain. Collectively, our current study indicates the importance of microglial Hv1-mediated ROS generation for microglia-astrocyte interaction and the development of neuropathic pain hypersensitivity.

## Materials and methods

### Animals and surgery

Mice were maintained in the animal facility at Rutgers University and Mayo Clinic. C57BL/6 J (Charles River), CX_3_CR1^GFP/+^, Hv1^−/−^ (C57BL/6 J background), and Hv1^−/−^/CX_3_CR1^GFP/+^ mice were used. Considering sex-dependent role of microglia in neuropathic pain [44], we used all male mice for the current study. The genotype was blind to the experimenters. Lumbar 4 spinal nerve transection (SNT) surgery was done in 7–9 weeks old mice as described before [12, 81]. SNT surgery was performed under 2% isoflurane anesthesia. An incision was made along the middle line of lumbar spine. Left side paraspinal muscles in front of pelvis bone were separated to expose the L5 transverse process. The L5 transverse process was removed to expose L4 spinal nerve. The L4 spinal nerve was separated and transected and removed 1–1.5 mm from one end. The wound was then irrigated with PBS and closed with #6 silk sutures for the muscles and #5 silk sutures for the skin. All animal experiment protocols were reviewed and approved by the Institutional Animal Care and Use Committee at Rutgers University and Mayo Clinic.

### Behavioral measurement

Mechanical allodynia was assessed by measuring the paw withdrawal threshold with a set of Von Frey filaments (0.04–2 g; North Coast medical). Mice were placed on an elevated metal grid. The filament was applied to the plantar surface in vertical angle for 1–2 s from the bottom. 50% withdrawal threshold values were determined using the up-down method.

Thermal hyperalgesia was assessed by measuring the paw withdrawal latency to radiant heat stimuli. Mice were placed in elevated chambers with Plexiglas floor and allowed to habituate for 20 min. The radiant heat source (IITC Inc life science) was applied to the center of the plantar surface in the hind paw for 4 times with at least 5 min intervals. The average withdrawal latency of the 4 trials was recorded as the response latency.

For the tail flick test, mice were restrained in a cylinder with tail hung out. The mice were then placed on the test panel with the tail covered a detection hole at a fixed distance to the tail root. The same radiant heat was focused on the tail at the detection hole position. Tail flick will expose the hole to the light and trigger the sensor to record the latency time. The tests were done for 4 times with at least 5 min intervals. The average withdrawal latency of the 4 trials was recorded as the flick response latency.

The rotarod tests were performed using a four-lane Rotarod apparatus (Med Associates Inc). The rota-rod speed started from 4 Round Per Minute (RPM) and uniformly accelerated to 40 RPM in 5 min. Each mouse was tested for 3 times with 5 min intervals for the first day and one time for the next day.

### Whole-cell patch clamp recording in microglia

Whole-cell patch clamp recording was performed as reported previously [82, 83]. Mice were anesthetized with isoflurane and coronal slices (300 μm) of spinal cord were prepared. After 1 h of recovery, brain slices were perfused with oxygenated artificial cerebrospinal fluid (ACSF) solution at 3–4 ml/min. NaCl was replaced with NaMeSO3 to eliminate potential Cl- currents. Whole-cell patch-clamp recordings in microglia were made using 5–10 MΩ glass pipettes filled with a TMA-based intracellular solution consisting of 100 mM TMA-MeSO3, 1 mM EGTA, and 100 mM MES (pH 5.5, 290–300 mOsm). The membrane potential was held at − 60 mV. Data were amplified and filtered at 2 kHz by a patch-clamp amplifier (Multiclamp 700B), digitalized (DIGIDATA 1440A), stored, and analyzed by pCLAMP (Molecular Devices, Union City, CA). Data were discarded when the input resistance changed > 20% during recording. A minimum of five cells from at least three different mice from the same litter were randomly selected for recording per condition.

### Western blot analysis

Lumbar 4–5 spinal dorsal horns were collected and protein was extracted. 50 μg of protein from each group was then loaded and separated by SDS-PADGE, transferred to a PVDF membrane, blocked with 5% skim milk in TBST, and incubated overnight with primary antibodies at 4 °C. Primary antibodies include, rabbit anti-Hv1 (1:2000; AHC-001, RRID:AB_10917155, Alomone labs) and GAPDH (1:1000; sc-32233, RRID:AB_627679, Santa Cruz Biotechnology). Membranes were incubated with horseradish peroxidase-conjugated goat anti-rabbit IgG (1:2000; 111-036-045, RRID:AB_2337943, Jackson ImmunoResearch Labs) and horseradish peroxidase-conjugated goat anti-mouse IgG (1:2000; 115-035-003, RRID:AB_10015289, Jackson ImmunoResearch Labs) for 1 h at room temperature. Membranes were then treated with West Pico substrate (34078, Thermo Fisher Scientific) and chemiluminescence signal was detected with a G:BOX Chemi XRQ gel doc (Syngene, Frederick, MD). Optical density of each band was then determined using Fiji, (NIH).

### Real-time qRT-PCR

Total RNA from isolated L4 spinal cord tissue was extracted using TRIzol reagent (Invitrogen). Real-time qRT-PCR was performed using a 7500 Real-Time PCR system (Applied Biosystems) with oligonucleotide primers. The primer sequences are listed as Table 1. The threshold cycle (Ct) of the GAPDH gene was used as a reference control to normalize the expression level of the target gene (ΔCt) to correct for experimental variation. Relative mRNA levels were calculated according to the 2^−ΔΔCt^ method. Real-time qRT-PCR experiments were performed at least three times, and the mean ± SEM values are presented unless otherwise noted.

**Table 1:** Primer sequences for qRT-PCR

cytokine	forward	reverse
IFN-γ	AGCGGCTGACTGAACTCAGATTGTAG	GTCACAGTTTTCAGCTGTATAGGG
IL-18	ACTGTACAACCGCAGTAATACGG	AGTGAACATTACAGATTTATCCC
IL-1β	CCAAAAGATGAAGGGCTGCT	TCATCAGGACAGCCCAGGTC
TNF-α	ATGCTGGGACAGTGACCTGG	CCTTGATGGTGGTGCATGAG
BDNF	AGGCACTGGAACTCGCAATG	AAGGGCCCGAACATACGATT
GAPDH	AACTCCCTCAAGATTGTCAGCAA	GGCTAAGCAGTTGGTGGTGC

### Drug administration

For intrathecal (i.t.) drug administration, mice were hand restricted and injected by direct lumbar puncture between L5 and L6 vertebrae of the spine, using a 10-μL Hamilton syringe (Hamilton Bonaduz AG) with a 31G needle. Successful insertion was indicated by tail flick response. DHE (10 μg in 5 μl ACSF, prepared with 100 mg/ml stock in DMSO), IFN-γ (100 ng in 5 μl ACSF), and IFN-γ neutralizing antibody (200 ng in 5 μl ACSF) were injected to awake mice at given time points after SNT. Sulforaphane (Calbiochem) was intraperitoneal (i.p.) treated. The stock solution was prepared in DMSO and diluted (1 mg/ml in 10% corn oil in PBS) just before use. BrdU (Sigma, 50 mg/kg in PBS) was i.p. injected at 30 min before SNT surgery and then daily in the following days with the same dose. The last injection was 4 h before the scarification.

### In situ ROS detection

Dihydroethidium (DHE), an oxidative fluorescent dye, was used to detect ROS in the spinal cord after SNT surgery. To avoid reduction of ROS fluorescence during immunostaining, DHE (Sigma, 10 μg in 5 μl ACSF) was injected by direct lumbar puncture between L5 and L6 vertebrae of the spine, using a 10-μL Hamilton syringe (Hamilton Bonaduz AG) with a 31G needle. Two hours after the DHE injection, the mice were anesthetized and perfused intracardially with 20 ml PBS followed by 20 ml of cold 4% paraformaldehyde (vol/vol) in PBS. The spinal cord was harvested similar to that for immunostaining and stored in dark. The samples were then cryocut within 48 h after perfusion fixation and imaged within 2 h after the slice mounting. DHE, DAPI signals, and the area of the microglial cell body were quantified with ImageJ software (US National Institutes of Health) by masking non-microglial cells. The oxidation of DHE in vivo can generate multiple fluorescent products with overlapping spectra, which may not be exclusively located in the nucleus.

### Fluorescent immunostaining

The mice were deeply anesthetized with isoflurane (5% in O_2_) and perfused transcardially with 40 ml PBS followed with 40 ml cold 4% PFA in PBS. The spinal cord was removed and post-fixed with the same 4% PFA overnight at 4 °C. The spinal cord was then transferred to 30% sucrose in PBS for at least 24 h. Spinal cord transverse Sects. (15 μm in thickness) were prepared on gelatin-coated glass slide with a cryocut microtome. The sections were blocked with 5% goat serum and 0.3% Triton X-100 (Sigma) in TBS buffer for 1.5 h. The sections were incubated overnight at 4 °C with primary antibody for rabbit-anti-Iba1 (1:1000, Wako), mouse-anti-BrdU (1:200, Sigma), mouse-anti-8-OHG (1:200, Abcam), rabbit-anti-p-p38 (1:400, cell signaling), mouse-anti-Nox2/gp91-phox (1:100, Santa Cruz), rat-anti-IFN-γ (1:100, eBioscience) rabbit-anti-BDNF (1:200, Alomone Labs Ltd, Israel). The sections were then incubated for 1.5 h at RT with corresponding secondary antibodies (Alexa Fluor 555 & 488, Invitrogen). The sections were mounted with Fluoromount-G (SouthernBiotech) and fluorescent images were obtained with a confocal microscope (LSM510, Zeiss). For BrdU staining, a pre-antigen retrieval process was made. Slices were immersed in 0.1 M citrate buffer (pH 6.0) for 10 min, keeping the temperature at 92–98 °C. After a wash with PBS for 5 min at RT, slices were incubated in 2 N HCl for 30 min and then washed in PBS for 10 min at RT. Regular staining process was undergone afterward.

Iba1 or BrdU positive cell densities and fluorescent signal intensity of 8-OHG and GFAP were quantified using ImageJ software (National Institutes of Health, Bethesda, MD). The intensity of ox-DHE, p-p38 were measured within GFP or Iba1 positive area, respectively. The area mask and intensity measurement were done using a Matlab (The MathWorks, Inc.) script. IFN-γ and Nox2/gp91-phox were measured within GFAP positive area and done under ImageJ as well.

### Statistics analysis

Quantification of fluorescent immunostaining results was done with Fiji version of ImageJ (Fiji, RRID:SCR_002285). Pain behaviors were analyzed using two-way ANOVA with multi comparisons to test for main effects between groups followed by post-hoc testing for significant differences. Two-group analysis utilized the Student’s t-test. Three to four group analyses utilized a one-way ANOVA design. Data are expressed as mean ± SEM. All statistical analyses were performed using GraphPad Prism 8 software (GraphPad Prism 8, RRID:SCR_002798). Level of significance is indicated with *p < 0.05, **p < 0.01, ***p < 0.001, ****p < 0.0001.

## Data Availability

The datasets are available from the corresponding author on reasonable request.
